# Exploring EFL primary school teachers’ behavioral intention towards digital game-based learning

**DOI:** 10.1371/journal.pone.0346229

**Published:** 2026-04-01

**Authors:** Yuting Wang, Deping Zou

**Affiliations:** 1 Faculty of Foreign Languages, Qilu Normal University, Jinan, China; 2 School of Education, Shanghai International Studies University, Shanghai, China; Golestan University, IRAN, ISLAMIC REPUBLIC OF

## Abstract

This study investigates the factors influencing English as a Foreign Language (EFL) primary school teachers’ behavioral intention (BI) to integrate Digital Game-Based Learning (DGBL) into their teaching practices. Despite digital gaming’s growing popularity as an educational tool, its application in primary school EFL instruction remains limited. To bridge this gap, the research employs a quantitative approach utilizing Partial Least Squares Structural Equation Modeling (PLS-SEM) within the context of the Technology Acceptance Model (TAM) to explore EFL teachers’ BI to implement DGBL. A sample of 500 EFL primary school teachers from Shandong Province, China, was surveyed. The study analyzed the relationships among five variables: attitude towards technology (ATT), perceived usefulness (PU), technology perception (TP), technology anxiety (TA), and BI. The results reveal that ATT and TP are significant predictors of teachers’ BI to employ DGBL. Conversely, TA does not exert an influence on ATT. The mediating role of ATT is demonstrated by the positive influence of PU and TP on it, which subsequently reinforces teachers’ BI to adopt DGBL. By contrast, TA does not exhibit a notable impact on ATT, suggesting a limited indirect effect of TA on their BI to engage with DGBL. These findings offer valuable insights for stakeholders interested in promoting the adoption of innovative technologies like digital games in primary education. The study concludes with practical implications for DGBL implementation and proposes potential avenues for future research.

## 1. Introduction

The role of play in learning, emphasized by scholars from Plato to Vygotsky, Piaget, and Rousseau, has long been recognized as essential for children’s intellectual, social, and emotional development [1, 2]. Digital game-based learning (DGBL) harnesses the engaging qualities of play, integrating the entertainment value of games with educational objectives to enhance student motivation and academic outcomes [3, 4]. Although digital games have become prominent pedagogical tools across various curricula [5–8], their integration within formal English as a Foreign Language (EFL) instruction remains limited, particularly at the primary school level [9].

Understanding teachers’ behavioral intention (BI), defined as their conscious plans to adopt specific technologies, is critical for facilitating the successful implementation of DGBL [10–17]. While the classic Technology Acceptance Model (TAM) posits that perceived usefulness (PU) and attitude (ATT) are primary predictors of BI, relying solely on these variables facilitates an incomplete understanding of DGBL adoption in primary education as it overlooks specific classroom nuances and distinct affective factors. Consequently, this study extends the TAM framework by incorporating Technology Perception (TP) and Technology Anxiety (TA) to capture pedagogical applicability and psychological apprehension, respectively. Integrating these factors is essential to comprehensively explore the BI of Chinese EFL primary school teachers, a research context that currently remains underexplored [18–21]. This research gap merits attention as a nuanced analysis of these factors yields empirically grounded recommendations for optimizing DGBL implementation.

Amidst global digital transformation, educational systems increasingly integrate digital games into learning environments [22]. By systematically examining the psychological and situational determinants influencing teachers’ BI, this research aims to enhance DGBL implementation and instructional quality within the Chinese context. Theoretically, this study extends the TAM framework by integrating TP and TA within the Chinese context. This model elucidates both the cognitive and emotional drivers underlying DGBL adoption, thereby refining existing TAM-based research by offering a more fine-grained understanding of these mechanisms in the Chinese primary EFL context. Practically, the findings provide policymakers and school administrators with targeted insights to refine support systems based on specific teacher variables. These insights highlight the necessity of addressing teacher apprehension and ensuring pedagogical fit, which in turn encourages more effective collaboration between educators and technology developers. Overall, this study underscores the need for concerted efforts among all stakeholders to leverage the potential of DGBL fully and sustainably within the educational system.

This study aims to examine the determinants influencing EFL primary school teachers’ BI to adopt DGBL through this extended TAM framework. It investigates the predictive relationships among ATT, PU, TP, TA, and BI in classroom practices. The research proposes ten hypotheses positing that ATT directly predicts BI while PU, TP, and TA function as pivotal antecedents that predict both teachers’ ATT and their BI. Furthermore, this study examines mediation pathways whereby ATT mediates the effects of PU, TP, and TA on teachers’ BI regarding DGBL implementation.

## 2. Literature review

### 2.1. Digital game-based learning

Games have historically provided structured experiential learning opportunities, significantly enhancing cognitive, social, and emotional development across cultures. In contemporary times, the rise of the digital native generation, familiar with diverse digital technologies, has transformed traditional learning paradigms, placing digital games at the forefront of innovative educational strategies [23, 24]. DGBL thus leverages these digital platforms, integrating motivational, cognitive, and social affordances within cohesive and engaging educational environments [25–27]. Consequently, investigating the effectiveness of digital games in education and optimizing their application according to specific contexts have become crucial research topics.

Research consistently highlights that core game mechanics such as immediate feedback, progressive challenges, and goal-oriented tasks are effective in promoting deep learner engagement and intrinsic motivation. These mechanics enable individualized learning pathways and foster essential higher-order thinking and problem-solving skills within collaborative or competitive contexts [28–31]. Unlike traditional educational models that often rely heavily on rote memorization and standardized instruction, DGBL supports learner-centered approaches, naturally appealing to students’ interests and encouraging active participation [32, 33].

Nevertheless, the effectiveness of DGBL is not universally guaranteed and is significantly shaped by contextual factors. International research underscores that successful implementation depends on access to suitable technology, alignment with curriculum standards, and cultural acceptance of game-based methods. This international perspective emphasizes personalized learning experiences and flexible curricular integration, common in Western educational settings, where teachers generally enjoy more autonomy to innovate pedagogically. However, the predominantly Western orientation of these research approaches may not directly translate into contexts with significant cultural and institutional differences, such as the educational environment in China.

In contrast, educational contexts in China present unique challenges and opportunities for DGBL integration. Chinese education traditionally emphasizes high-stakes testing and teacher-centered instruction, potentially limiting the initial adoption of student-centered innovations like DGBL. Despite structural constraints, Chinese educational reforms increasingly recognize the need to foster communicative competence and critical thinking, creating a fertile environment for introducing DGBL. However, critical comparisons between international and Chinese contexts illuminate the necessity for adapting DGBL approaches. While Western frameworks prioritize learner autonomy and flexible integration, the Chinese educational landscape requires a more structured approach to fit local pedagogical norms. Thus, effective implementation involves creating content that resonates with students’ background knowledge to enhance motivation [34, 35]. Recent studies illustrate this adaptation strategy, such as the use of digital escape-room games that utilize familiar narratives like interacting with a Kung Fu master alongside tasks reflecting local orthographic reasoning patterns [35]. These examples demonstrate that successful DGBL integration relies on grounding game mechanics in familiar contexts to address specific educational objectives and reduce learner anxiety [6, 26, 34, 36].

In summary, although international research broadly confirms the educational potential of DGBL, its practical application in specific contexts, such as Chinese primary EFL classrooms, necessitates careful attention to cultural and institutional particularities. By critically integrating global insights with local educational realities, DGBL can effectively enhance language learning outcomes, support educational reforms, and sustainably foster learner engagement and motivation.

### 2.2. DGBL in EFL learning

Building upon the theoretical and empirical foundations of DGBL, a growing body of research has examined its integration specifically within EFL educational contexts [37]. This interest is particularly evident in non-English-speaking regions such as China, where English proficiency is both a crucial educational goal and an important social benchmark [34, 38, 39]. The incorporation of DGBL into EFL teaching represents more than just a technological advancement; it reflects sociocultural and pedagogical demands for more engaging, effective, and learner-centered instructional approaches.

Recent research has identified several overarching themes that illustrate the broad impacts and potential of DGBL in EFL contexts. Firstly, DGBL has consistently shown potential in increasing learner motivation, engagement, and positive attitudes toward language learning, outcomes integral to achieving sustained progress in EFL settings [34, 40–44]. By embedding language learning tasks within interactive and authentic game scenarios, DGBL translates abstract linguistic concepts into tangible, experiential activities, facilitating deeper understanding and skill application [35, 36]. Moreover, motivational game features, such as immediate feedback, achievable challenges, and social interactions, leverage both intrinsic and extrinsic motivation, thereby enhancing academic performance and language retention [45, 46].

Secondly, international research underscores DGBL’s potential to benefit a diverse range of learners, including those with lower proficiency or traditionally marginalized within classroom settings. Empirical studies conducted across different cultural contexts illustrate that DGBL effectively fosters self-regulated learning, collaborative problem-solving, and social engagement, all essential for successful language acquisition [47, 48]. Specifically, within the Chinese educational landscape, DGBL has demonstrated effectiveness in mitigating language anxiety and enhancing learners’ willingness to communicate, which are critical barriers prevalent in conventional EFL classrooms characterized by exam-oriented and rote learning practices [34].

Nevertheless, the successful integration of DGBL in EFL contexts hinges on several local factors, including technological infrastructure, curriculum alignment, and teacher digital literacy. In Western educational systems, DGBL implementation typically emphasizes personalized learning experiences and flexible curriculum integration. Conversely, in China and other Asian contexts, DGBL often functions as a complementary strategy aimed at improving communicative competencies and engagement within systems predominantly driven by standardized testing [34, 44]. This context underscores the need to ensure digital tools are adapted to support rather than disrupt curricular standards. Recent empirical evidence demonstrates how specific alignments optimize educational effectiveness. For instance, Duolingo activities have been successfully integrated by matching vocabulary and listening modules with local curricular themes to ensure complementarity with traditional instruction [34]. This example highlights that successful DGBL implementation in EFL classrooms requires synchronizing digital tasks with mandated learning objectives to meet the specific needs of Chinese learners.

In conclusion, existing research converges on the consensus that DGBL represents a highly promising instructional approach for EFL education, particularly when implementation strategies are carefully attuned to local pedagogical and sociocultural conditions. Through integrating motivational, cognitive, and social dimensions, DGBL can significantly enhance language learning outcomes, support educational equity, and promote sustained learner engagement and lifelong learning.

### 2.3. EFL teachers’ acceptance of DGBL

Although DGBL provides significant pedagogical advantages and increasing accessibility for students, its successful integration into EFL classrooms fundamentally depends on teachers’ acceptance and their professional agency [44, 46]. As primary facilitators of educational innovation, teachers’ attitudes, self-efficacy, and BI critically determine whether DGBL transitions effectively from experimental trials to mainstream teaching practices [47, 49].

Synthesizing international and Chinese research, several interconnected themes emerge. Many EFL teachers globally recognize the positive impacts of DGBL on student engagement, motivation, language proficiency, and improved classroom dynamics [34, 44, 50]. Teachers who exhibit favorable attitudes toward DGBL often emphasize its ability to promote communicative competence, create authentic collaborative learning environments, and address diverse learner needs [35, 36]. High acceptance typically correlates with familiarity and positive past experiences with educational technologies, supportive institutional policies, and robust professional development programs [47, 50–52].

Conversely, teachers’ acceptance varies notably due to differences in national, institutional, and personal contexts. In examination-oriented educational systems like China, teachers frequently express concerns about technical difficulties, classroom management issues, perceived superficiality or excessive competitiveness in digital games, and doubts regarding their instructional efficacy and capacity for formative assessment [35, 47, 48]. Additionally, teachers may worry that digital games could divert students from curricular objectives or encourage non-educational behaviors, particularly when students lack developed self-directed learning skills [36]. These reservations are intensified by limited information and communication technology (ICT) proficiency, heavy teaching loads, inadequate training opportunities, and insufficient institutional incentives [47, 49, 53, 54].

Comparative studies further reveal that Chinese teachers’ acceptance of DGBL is influenced not merely by technological or pedagogical factors but by broader sociocultural dynamics, including the emphasis on high-stakes examinations, traditional instructional approaches, and parental expectations, which may not align well with game-based learning methods [34]. In contrast, educators in Western contexts often experience more flexibility in employing learner-centered pedagogies, though similar challenges such as time constraints and resource limitations also exist [45].

Therefore, promoting effective DGBL implementation in EFL contexts requires addressing these multifaceted challenges through targeted professional development, enhancing digital literacy, cultivating collaborative educational cultures, and localizing digital game content to align with curricular and cultural expectations [35, 36, 47]. Understanding teachers’ complex and context-specific acceptance and behavioral intentions is essential for scaling DGBL and fully realizing its educational potential in EFL classrooms [55, 56].

## 3. Theoretical framework and hypotheses development

**Fig 1:**
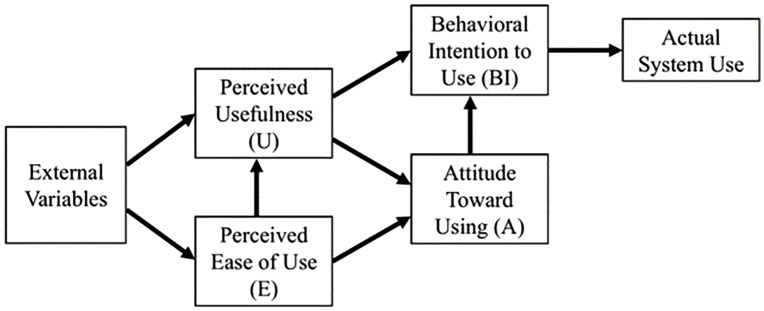
Original TAM proposed by Davis (1989), redrawn by the authors based on Davis (1989) and Chen (2023).

To investigate the behavior of technology users, Davis et al. [57] synthesized the initial Theory of Reasoned Action (TRA) from psychological research and integrated it with information systems usage, culminating in the creation of the original Technology Acceptance Model (TAM) [8, 18], as illustrated in Fig 1 sourced by Chen [8]. TAM indicates that perceived usefulness (PU), perceived ease of use (PEOU), attitudes toward utilizing technology, and BI to use that technology are interconnected. The TAM offers insights into the factors that determine computer acceptance, including users’ BI, attitudes, PU, and PEOU related to e-learning systems, applicable to a wide array of end-user computing technologies and different user demographics [18]. Within educational contexts specifically, the TAM constitutes a highly regarded and empirically validated framework for analyzing teachers’ behaviors regarding the adoption of innovative technologies [18]. Therefore, the present study employs TAM to examine the BI of EFL primary teachers to embrace and utilize DGBL. Throughout its evolution, the TAM has undergone significant refinement and extensions [58–62] to facilitate empirical examination and elucidation of factors influencing an individual’s acceptance, rejection, or sustained use of emergent technologies [3]. The model has been extensively validated empirically, demonstrating its competency to explicate an individual’s task performance by taking into account their BI to engage in that specific task. In addressing challenges associated with comprehending the predictors of TAM variables, the model has been expanded to encapsulate novel factors and variables exerting significant influence [63]. Based on previous studies, Abdullah and Ward [59] have synthesized the most frequently employed external factors for TAM, including self-efficacy, subjective norm, enjoyment, computer anxiety and experience. Ayanwale [64] has utilized external factors in the context of TAM, notably confidence in teaching technology (CTT) and technology anxiety (TA). Additionally, Adelana et al. [3] have incorporated diverse external elements into their analysis of TAM, including technology for social good (TSG), technology perception (TP), technology anxiety (TA), and technology readiness (TR).

**Fig 2:**
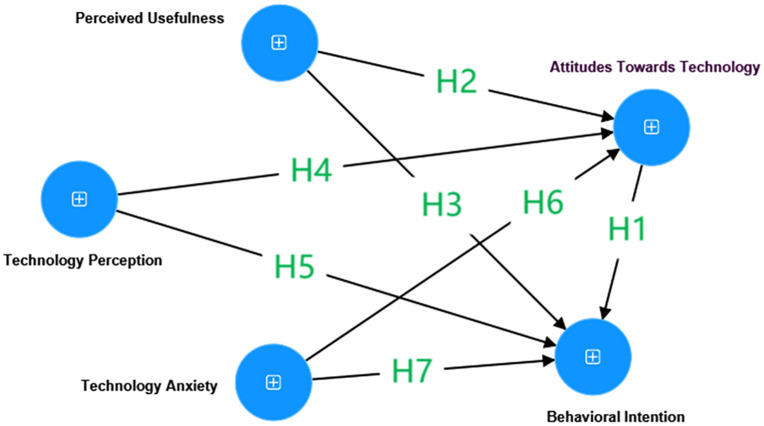
Hypothesized Structural Model for the latent variables.

This investigation delves into the predictive factors underpinning the acceptance and application of DGBL within education among EFL primary school teachers. By leveraging the extended TAM, this study analyzes constructs utilizing factors, including ATT, PU, TP, TA, and their impact on the BI of EFL primary school teachers to integrate DGBL into their teaching practices. A recent meta-analysis conducted by Scherer et al. [65] identified the TAM as a robust framework that proposes both direct and indirect mechanisms influencing teachers’ technological integration [38]. This analysis not only aids in enhancing the model’s explanatory power and predictive accuracy but also provides a scientific basis for improving teachers’ experience and technology acceptance [38]. Simultaneously, it drives further development and refinement of theoretical frameworks. Examining these mediating effects provides deeper insights into the psychological processes influencing teachers’ acceptance of new technologies, offering a theoretical and empirical foundation for designing effective interventions [65], thereby offering empirical guidance for designing effective technological interventions. Therefore, this study incorporates mediation analysis to explore indirect relationships among specific constructs. Specifically, it scrutinizes how ATT mediates the respective effects of PU, TP, and TA on BI of EFL primary school teachers to use DGBL. BI is employed as the dependent variable instead of actual usage due to the potential for teachers’ DGBL implementation to be influenced by extraneous barriers, such as those imposed by institutional and district-level policies [66]. The conceptualized model and proposed hypotheses are shown in Fig 2.

### 3.1. Attitude towards technology (ATT)

An individual’s BI is shaped by their attitude towards the specific behavior [18]. Attitude refers to an individual’s positive or negative emotional disposition towards engaging in a particular behavior [67]. It encompasses a subjective evaluation of a psychological entity, characterized by dimensions such as favorable versus unfavorable, enjoyable versus unenjoyable, or appealing versus unappealing [68]. Teachers’ attitudes toward technology integration serve as a barometer for their propensity to harbor positive feelings and opinions regarding the utilization of technology [69]. Consequently, empirical evidence substantiates that attitude is a salient variable that influences an individual’s BI to engage with technology [69–73]. According to Ertmer et al. [74], teachers’ positive attitudes toward integrating technology are crucial for the successful facilitation of effective learning experiences. Based on the literature, this study hypothesizes that:

**H1.** ATT predicts BI of EFL primary school teachers to use DGBL in class.

### 3.2. Perceived usefulness (PU)

PU is defined as the extent to which an individual believes that the utilization of a specific system would enhance their job performance [75]. The underlying rationale for this proposition is that within a work environment, if individuals anticipate that technology will augment their job performance, their inclination to use the technology will supersede what can be solely attributed to their attitude towards the technology itself [76]. In the context of education, perceived usefulness may pertain to teachers’ willingness to use technology based on their perception of its potential to bolster their teaching efficacy. Empirical research indicates that perceived usefulness exerts a significant influence on BI to engage with a particular technology [18, 65, 77–79]. Furthermore, according to the TAM model [60, 64, 75], ATT functions as a mediating factor that influences individuals’ PU toward technology, thereby shaping their BI in using technological tools and innovations. Because of this, this study posits that:

**H2.** PU predicts ATT of EFL primary school teachers to use DGBL in class.

**H3.** PU predicts BI of EFL primary school teachers to use DGBL in class.

### 3.3. Technology perception (TP)

Technology Perception (TP) refers to teachers’ specific judgment on the effectiveness and feasibility of using digital games within the classroom environment. Distinct from PU, which centers on professional productivity, and ATT, which reflects a general positive or negative disposition, TP focuses specifically on the pragmatic applicability of the technology to enhance student outcomes and manage instructional dynamics. Possessing an accurate and comprehensive technology perception positively influences the adoption behaviors of teachers [80]. Although there is a plethora of studies on general technology perception [81–84], their findings may not be pertinent to the specific context of employing DGBL within formal educational settings. Burch and Mohammed [85] assert that the advent of novel technologies inevitably catalyzes innovative and adapted methodologies for learning and instruction. Consequently, teachers must evaluate not just the tool’s utility, but its compatibility with the complex realities of the classroom. Teachers’ perceptions of this pedagogical fit are pivotal in their determination to employ the tool, which subsequently impacts their BI to use the technology [3]. Meanwhile, Fazio and Williams [86] posit that behavior originates from individuals’ attitudes, which are formed based on their perceptions of the object within specific contexts. In line with this framework, perception serves as a cognitive antecedent; thus, specific judgments regarding classroom effectiveness can significantly shape teachers’ attitude towards using the technology. This implies a sequential pathway where favorable classroom perceptions foster positive attitudes, which subsequently drive behavioral intention. However, scant research has empirically tested this causal pathway. Hence, this study proposes that:

**H4.** TP predicts ATT of EFL primary school teachers to use DGBL in class.

**H5.** TP predicts BI of EFL primary school teachers to use DGBL in class.

### 3.4. Technology anxiety (TA)

Individual anxiety associated with technology predominantly centers on the psychological state of users, particularly their ambivalence and reservations concerning their competence and readiness to adeptly employ technology-based instruments [87]. According to Troisi et al. [88], TA refers to a multifaceted constellation of emotions encompassing apprehension, incertitude, and fears that arise in conjunction with the application and acquisition of technological proficiencies. TA can be assessed both as a predictor of individuals’ behavior and a salient factor impacting their BI [87]. However, the variable of teacher anxiety, specifically within the context of technological transformation and its repercussions on teacher emotional responses, constitutes an underexplored domain that has received relatively sparse research attention [89]. Numerous factors contribute to the manifestation of TA among teachers. These include their proclivity towards conventional pedagogical methodologies, which may not harmonize with the integration of technology in educational settings, their unease with unfamiliar technological knowledge and skill sets, and the relatively diminished perception skills towards technology exhibited by experienced teachers [90]. Moreover, scholarly literature presents divergent perspectives on the relationship between anxiety and attitude. Certain studies have treated anxiety as a form or component of attitude [91, 92], whereas others have delineated them as distinct variables [93, 94]. Evidently, this study has bifurcated the two terms into separate variables in order to explore the relationship between TA and ATT. Based on the extant literature, this research focuses on:

**H6.** TA predicts ATT of EFL primary school teachers to use DGBL in class.

**H7.** TA predicts BI of EFL primary school teachers to use DGBL in class.

This study also explored the following indirect effect relationships:

**H8.** ATT mediates the relationship between PU and BI of EFL primary school teachers to use DGBL in class.

**H9.** ATT mediates the relationship between TP and BI of EFL primary school teachers to use DGBL in class.

**H10.** ATT mediates the relationship between TA and BI of EFL primary school teachers to use DGBL in class.

## 4. Method

### 4.1. Participants

This study analyzed a valid sample of 500 in-service EFL primary school teachers from Shandong Province, China, recruited through a convenience sampling method. All participants engaged voluntarily, and their data were analyzed and are presented in Table 1. Among the collected responses, female teachers comprised 76.6% of the cohort. The age demographic of the teachers spanned from 21 to 55 years, with the most populous segment being those aged between 26 and 35 years, accounting for 41.8% of the respondents. This distribution suggests a predominance of individuals from the techno-mobile age group within the context of the fourth industrial revolution. In terms of pedagogical tenure, the majority of these teachers (55.6%) possess teaching experience ranging from 1 to 10 years, indicating a certain level of teaching proficiency and a greater adaptability to advanced technology compared to those with over 20 years of experience (33.6%).

**Table 1:** Participants’ demographic profile.

Variable	Categories	Frequency	Percent
Gender	Female	383	76.6
	Male	117	23.4
Age	21–25	56	11.2
	26–30	100	20.0
	31–35	109	21.8
	36–40	59	11.8
	41–45	35	7.0
	46–50	66	13.2
	50–55	75	15.0
Pedagogical tenure	1–5	149	29.8
	6–10	129	25.8
	11–15	27	5.4
	16–20	27	5.4
	21–25	35	7.0
	26–30	67	13.4
	30 and above	66	13.2

### 4.2. Survey instrument

This survey, conscientiously adapted from the pre-existing, validated surveys [65, 83], was segmented into the following two sections. The first section of the survey, adapted from Khukalenko et al. [83], consisted of seven multiple-choice questions, focusing on background data, including demographic information, teaching methods, availability of DGBL equipment, availability of IT support, and frequency of DGBL usage. The second section evaluated parameters to gauge teachers’ BI to incorporate DGBL within classroom settings. TP, as one of the variables, contained 12 items adapted from Khukalenko et al. [83]. In addition, the items in the other four variables, including ATT, PU, TA, and BI were adapted from Ayanwale et al. [65]. ATT, PU, and TA each incorporated 3 items, whereas BI included 5 items. Altogether, a total of 26 items were incorporated into the survey instrument. Responses were recorded on a five-point Likert scale: 1 – *Strongly Disagree*, 2 – *Disagree*, 3 – *Neutral*, 4 – *Agree*, 5 – *Strongly Agree*. The original scales were adapted into Chinese using the translation and back-translation method to guarantee linguistic and cultural validity. Regarding data integrity, submissions were restricted to one per IP address, and reverse-coded items (e.g., TP2) were included to monitor participant attention. Subsequent analysis confirmed that all constructs demonstrated satisfactory internal consistency with Cronbach’s alpha and Composite Reliability values exceeding recommended thresholds. Detailed reliability and validity metrics are explicitly reported in the Results section (see Table 2).

**Table 2:** Convergent validity and reliability of the constructs.

Variable	Item Loading	CA	CR	AVE	VIF
ATT1	0.825	0.766	0.865	0.681	1.555
ATT2	0.815				1.529
ATT3	0.836				1.594
PU1	0.849	0.874	0.909	0.665	1.626
PU2	0.822				1.554
PU3	0.802				1.500
TP1	0.755	0.765	0.864	0.680	1.874
TP10	0.774				1.974
TP11	0.791				2.032
TP12	0.817				2.231
TP2	0.762				1.919
TP7	0.753				1.913
TP8	0.778				1.999
TP9	0.793				2.122
TA1	0.872	0.851	0.909	0.770	2.151
TA2	0.877				1.995
TA3	0.883				2.104
BI1	0.807	0.907	0.925	0.606	1.908
BI2	0.819				2.029
BI3	0.817				1.958
BI4	0.808				2.016
BI5	0.827				1.994

ATT, attitude towards technology; PU, perceived usefulness; TP, technology perception; TA, technology anxiety; BI, behavioral intention; item loading >0.60; CA > 0.70; CR > 0.70; AVE > 0.5; VIF < 5.0.

### 4.3. Data collection

Data for this study were amassed using Wen Juan Xing, an online platform similar to Amazon Mechanical Turk, SurveyMonkey, or Cloud Research, which facilitates questionnaire formulation and survey administration. The survey link was disseminated to EFL primary school teachers via WeChat, a prevalent social media platform invaluable for survey distribution and collection due to its extensive user base and convenience; this link remained accessible from February 15th to March 18th, 2024 (approximately four weeks) prior to closure for data extraction. Participants were instructed to complete the survey on their mobile phones. Ethical oversight was ensured through formal approval from the Qilu Normal University Research Ethics Review Committee (Approval No. xsllsc2024−008). Prior to survey access, participants were presented with an electronic informed consent protocol on the Wen Juan Xing platform, requiring review of ethical disclosures and selection of “Agree” to proceed or “Disagree” to terminate participation (full text in S1 Appendix). Teachers were explicitly apprised that participation was voluntary and necessitated their consent, with abstention entailing no repercussions; they were additionally reassured of data confidentiality and protection before engaging with the survey form furnished by departmental representatives. Consequently, consent was procured preceding survey completion, and no ethical concerns emerged during data collection procedures, as confirmed through ongoing monitoring of participant engagement patterns and response integrity.

### 4.4. Data analysis

The study employed the quantitative analysis method of Partial Least Squares Structural Equation Modeling (PLS-SEM) through SmartPLS 4.1.0.9 software, concentrating on variance analysis to test the proposed model involving exogenous variables (perceived usefulness, technology perception, technology anxiety) and endogenous variables (attitude towards technology, behavioral intention) [95]. This method was selected for its flexibility in analyzing the interplay between hypotheses and empirical data (full data in S2 Appendix), effectively facilitating hypothesis development [96]. In addressing cultural and institutional dimensions, three control strategies were implemented. First, regionally homogeneous sampling of Shandong Province ensured uniform cultural traditions and policy standards; second, standardized measurement instruments under the TAM framework captured key variables incorporated in path analysis; third, multi-group analysis via PLS-SEM methodology empirically validated contextual effects. These approaches collectively address validity requirements concerning cultural-institutional dimensions.

The evaluation of the structural equation model consisted of two steps. Firstly, the measurement model was examined to assess the construct validity and reliability of each questionnaire item. Confirmatory composite analysis (CCA) was initially employed to validate the research instruments and factor structures. To ensure measurement validity, CCA incorporated item loadings, Cronbach’s alpha (CA), composite reliability (CR), average variance extracted (AVE), and the HeteroTrait-MonoTrait ratio (HTMT). Secondly, an exhaustive analysis of the structural model was conducted to evaluate the research hypotheses and explore the relationships among latent variables, such as ATT, PU, TP, TA, and BI to use DGBL. The study also examined the mediating effects of ATT on BI through PU, TP, and TA. In conclusion, a range of advanced statistical techniques was employed to test the research hypotheses and evaluate the relationships between latent variables associated with the behavioral intentions of using DGBL.

## 5. Results

### 5.1. Measurement model analysis

Each construct and its indicators are defined in the measurement model, while Fig 2 depicts the hypothesized structural model among ATT, PU, TP, TA, and BI.

Table 2 presents the item loadings, reliability, and convergent validity of the constructs employed in the study. The measurement model incorporated 22 items loaded under their respective latent variables, with low-factor-loading items (like TP3, TP4, TP5, and TP6) being removed as their loadings fell below the 0.60 threshold, indicating inadequate representation of the core “TP” construct and potential measurement invalidity. This enhances the model’s psychometric quality. The final outer loadings ranged from 0.753 to 0.883, surpassing the endorsed recommended threshold of 0.60 for PLS-SEM models [97]. Furthermore, the collinearity issue was assessed using variance inflation factors (VIF), where a value below 3 is considered ideal and below 5 is generally acceptable [98]. The VIF values ranged from 1.500 to 2.231, all beneath the recommended threshold. Moreover, to confirm the study’s convergent validity, Cronbach’s alpha (CA) and Composite Reliability (CR) values were calculated for each latent variable, meeting the threshold of 0.70 [99,100]: ATT (CA = 0.766, CR = 0.865), PU (CA = 0.874, CR = 0.909), TP (CA = 0.765, CR = 0.864), TA (CA = 0.851, CR = 0.909), BI (CA = 0.907, CR = 0.925). Additionally, average variance extracted (AVE) values were calculated for each latent variable, ranging from 0.606 to 0.770. All AVE values exceeded the recommended threshold of 0.50, further indicating strong convergent validity, as supported by Sarstedt et al. [100].

Furthermore, the study examined discriminant validity using the HeteroTrait-MonoTrait (HTMT), where a high discriminant validity implies that a construct captures unique aspects not captured by alternative measures [95]. Three established validation approaches were systematically evaluated in SmartPLS 4.1.0.9: the Fornell-Larcker criterion, cross-loading assessment, and the HTMT criterion. However, the HTMT criterion has emerged as the preferred method for evaluating discriminant validity due to its superior performance compared to the Fornell-Larcker criterion and cross-loading approach [101], which has recently become the primary criterion for assessing discriminant validity. An HTMT ratio below 0.90 indicates that the correlation between different constructs is significantly inferior to the correlation within each construct, signifying sound discriminant validity [65]. The discriminant validity results are presented in Table 3, where all constructs in the model exhibited HTMT ratios below the threshold of 0.90, demonstrating that the variables effectively measure their respective constructs.

**Table 3:** Discriminant validity- HeteroTrait-MonoTrait ratio.

Construct	ATT	BI	PU	TA	TP
ATT					
BI	0.727				
PU	0.518	0.468			
TA	0.228	0.362	0.180		
TP	0.605	0.572	0.366	0.274	

ATT, attitude towards technology; BI, behavioral intention; PU, perceived usefulness; TA, technology anxiety; TP, technology perception; HTMT <0.90.

### 5.2. Structural model analysis

**Fig 3:**
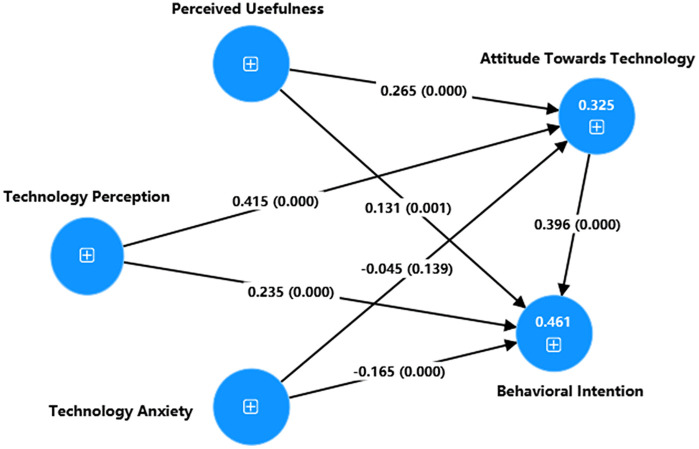
Path coefficient of the measurement model. p-values, numbers in parentheses; R^2^, numbers inside the dependent variables.

The study examined the direct relationship between the adopted exogenous variables and the criterion variable and tested the hypothesized relationship between these variables [98, 102]. The fundamental data depicting the relationship between these variables are illustrated in Fig 3. To enhance accessibility, Table 4 reports direct path coefficients with interpretive guidance: standardized path coefficient (β) indicates relationship strength (higher absolute values indicate stronger effect) and direction (sign indicates positive/negative association); sample mean (M) represents the central tendency of bootstrap estimates (closer to β indicates stable estimation); standard deviation (SD) measures dispersion of bootstrap estimates (lower values < 0.05 indicate high precision); t-values > 1.645 confirm statistical significance for our tests; 5%−95% confidence intervals excluding zero denote significant effects; and p-values < 0.05 establish statistical reliability. These metrics collectively enable robust hypothesis evaluation. (H1), ATT → BI to use DGBL (β = 0.396, t = 7.337, C.I = 0.307–0.484, p-value < 0.001) (H2), PU → ATT (β = 0.265, t = 6.993, C.I = 0.201–0.327, p-value < 0.001) (H3), PU → BI to use DGBL (β = 0.131, t = 3.208, C.I = 0.064–0.199, p-value = 0.001) (H4), TP → ATT (β = 0.415, t = 11.217, C.I = 0.354–0.475, p-value < 0.001) (H5), TP → BI to use DGBL (β = 0.235, t = 5.938, C.I = 0.171–0.301, p-value < 0.001) (H6), TA → ATT (β = −0.045, t = 1.804, C.I = −0.115–0.023, p-value = 0.139) (H7), TA → BI to use DGBL (β = −0.165, t = 4.671, C.I = −0.224–0.108, p-value = 0.000). Among the seven hypotheses examined in Table 4, six were found to be significant (H1-H5, H7), while one was deemed insignificant (H6). Additionally, as shown in Table 4, the R-squared value represents the proportion of variance in the dependent variable explained by the predictors. With R² values of 0.325 for ATT and 0.461 for BI, these indicate practically significant predictive power where independent variables explain substantial variance in both constructs. The 32.5% explanation of ATT variance reveals critical psychological drivers, while the 46.1% explanation of BI variance demonstrates strong predictability of adoption decisions. Collectively, the findings confirm that these variables directly support DGBL adoption intentions, providing actionable pathways for implementing DGBL in EFL classrooms.

**Table 4:** Direct path coefficient for tested model.

Relationships	β	M	SD	t-value	5%	95%	p-values	Remarks
H1: ATT - > BI	0.396	0.396	0.054	7.337	0.307	0.484	0.000	Supported
H2: PU - > ATT	0.265	0.265	0.038	6.993	0.201	0.327	0.000	Supported
H3: PU - > BI	0.131	0.131	0.041	3.208	0.064	0.199	0.001	Supported
H4: TP - > ATT	0.415	0.416	0.037	11.217	0.354	0.475	0.000	Supported
H5: TP - > BI	0.235	0.236	0.040	5.938	0.171	0.301	0.000	Supported
H6: TA - > ATT	−0.045	−0.045	0.042	1.804	−0.115	0.023	0.139	Not supported
H7: TA - > BI	−0.165	−0.166	0.035	4.671	−0.224	−0.108	0.000	Supported
	R-square							
ATT	0.325							
BI	0.461							

ATT, attitude towards technology; PU, perceived usefulness; TP, technology perception; TA, technology anxiety; BI, behavioral intention; β, standardized path coefficient; M, sample mean; SD, standard deviation; T = t > 1.645; p < 0.05.

The study also explored the mediating effects of ATT on the influence of PU, TP, and TA on BI to use DGBL. Path coefficients of the mediators are presented in Table 5. The findings reveal that (H8) PU positively and significantly influences ATT, subsequently impacting the BI to use DGBL (β = 0.105, t = 5.762, p-value < 0.001). This suggests that EFL primary school teachers’ favorable perception of technology is likely to enhance their willingness to use DGBL. Likewise, in accordance with (H9), TP has a notable and beneficial impact on ATT, resulting in an elevation of the intention to use DGBL ((β = 0.165, t = 6.121, p-value < 0.001)). This implies that teachers who perceive DGBL as beneficial and applicable in their teaching practices develop a positive attitude towards learning this technology, thereby boosting their inclination to incorporate it in the classroom. Intriguingly, (H10) TA does not exhibit a significant impact on teachers’ ATT, potentially not affecting their BI to use DGBL (β = −0.018, t = 1.079, p-value = 0.140). This suggests that EFL primary school teachers who feel anxious about technology may not necessarily have a negative attitude towards its use; therefore, their intention to implement it in the classroom is not diminished.

**Table 5:** Indirect effect relationship.

Relationships	β	M	SD	t-value	p-values	Remarks
H8: PU - > ATT - > BI	0.105	0.104	0.018	5.762	0.000	Supported
H9: TP - > ATT - > BI	0.165	0.165	0.027	6.121	0.000	Supported
H10: TA - > ATT - > BI	−0.018	−0.018	0.017	1.079	0.140	Not supported

ATT, attitude towards technology; PU, perceived usefulness; TP, technology perception; TA, technology anxiety; BI, behavioral intention; β, standardized path coefficient; M, sample mean; SD, standard deviation; T = t > 1.645; p < 0.05.

## 6. Discussion

In alignment with established TAM research [69, 73, 77, 103], the results confirm that teachers’ ATT serve as a primary driver of their intention to adopt DGBL. Positive attitudes correlate with greater motivation to acquire relevant skills, whereas negative dispositions correspond with instructional resistance. Similarly, PU is validated as a critical antecedent that directly predicts both teachers’ ATT and their BI [65, 104]. This confirms that when teachers perceive DGBL as a tool that enhances professional efficiency and student outcomes, they develop both a favorable regard for the technology and a higher likelihood of implementation. Beyond these direct effects, the relationship between PU and BI is significantly mediated by ATT. This mediation pathway suggests that the utility of DGBL fosters adoption by first cultivating a positive psychological disposition, which then translates theoretical value into practical classroom usage. These findings underscore the necessity of emphasizing pedagogical utility in professional development to systematically foster the favorable attitudes required for sustainable DGBL integration.

The findings concerning TP extend the understanding of DGBL adoption by validating its significant influence on both ATT and BI [54, 105]. The results indicate that when teachers perceive DGBL as a tool that genuinely benefits student outcomes and is manageable within the instructional setting, they are more likely to develop favorable attitudes and a direct intention to adopt it. Furthermore, the mediation analysis reveals that ATT serves as a crucial mechanism linking TP to BI. This implies a sequential pathway where the recognition of micro-level pedagogical fit first cultivates a positive evaluative disposition, which subsequently transforms into the BI to implement the technology. Consequently, successful DGBL integration depends on professional development that explicitly addresses classroom-level concerns, ensuring teachers perceive the technology as both educationally effective for students and practically feasible to manage.

The results regarding TA demonstrate that it negatively predicts teachers’ BI [106, 107]. It suggests that high levels of apprehension create a direct psychological barrier that discourages teachers from adopting DGBL in their classrooms. However, no significant relationship was found between TA and ATT; as a result, the mediating role of ATT was not supported. This finding implies that teachers’ nervousness regarding technical difficulties does not necessarily diminish their positive evaluation of DGBL’s educational value. While they may acknowledge the benefits of DGBL and maintain a positive attitude [108, 109], their technical apprehension acts as a direct hurdle to implementation, bypassing their attitudinal assessment.

## 7. Conclusion

This study employed an extended TAM to investigate BI among EFL primary school teachers regarding DGBL adoption. Focusing on ATT, PU, TP, and TA, the research found that ATT was the strongest predictor of BI; PU and TP influenced both ATT and BI; and TA negatively affected BI while remaining unrelated to ATT. The influence of PU and TP on BI was mediated by ATT, whereas the influence of TA on BI was not. Overall, the study refines how TAM variables operate in primary EFL contexts and offers practical levers to support DGBL integration.

### 7.1. Implications

This research validates the extended TAM framework within primary EFL education in China, offering empirical evidence on the psychological and contextual determinants of DGBL adoption. Theoretically, the study confirms the robustness of classic TAM constructs while expanding the model to include TP and TA, elucidating both cognitive and emotional drivers of teacher behavior. Practically, the strong influence of PU and TP indicates that successful adoption depends primarily on pedagogical fit and curricular alignment rather than mere entertainment value. Crucially, the finding that TA hinders implementation despite positive attitudes reveals that the primary barrier is anxiety rather than a lack of conviction. This implies that professional support facilitates adoption not by further persuading teachers of DGBL’s value, but by mitigating anxiety through user-friendly technical assistance and low-stakes practice. Ultimately, bridging the gap between teacher willingness and actual usage requires that policy resources be directed specifically toward these anxiety-reducing mechanisms.

### 7.2. Limitations and future directions

Although this study provides valuable insights, several limitations should be acknowledged to properly contextualize the findings. Methodologically, the broad age range and variation in prior technology exposure may have shaped participants’ cognitive and emotional responses, and disciplinary differences could affect how constructs were appraised. These factors introduce heterogeneity that may influence effect sizes. Empirically, reliance on self-reported data introduces a risk of social desirability bias where participants may subconsciously inflate their positive responses. Practically, this implies that the observed behavioral intention reflects an idealized willingness that may be higher than actual usage levels in real-world classrooms. Furthermore, the reliance on a convenience sample recruited via online platforms limits the broader generalizability of the findings. This sampling approach likely favored teachers who are already familiar with digital tools, suggesting that the results represent a more digitally active group rather than the entire teaching population. Contextually, the exclusive focus on EFL primary school teachers in Shandong Province, China, limits generalizability; findings should be interpreted conservatively and adapted to local policies, resources, and cultures before broader application.

To propel the field forward, future research should implement stratified sampling contingent upon technological proficiency assessments to enhance data objectivity. Researchers should then introduce interventions through workshops enabling direct experiential learning of digital games’ pedagogical benefits, while concurrently employing mixed-methods approaches integrating quantitative surveys with qualitative interviews to explore instructional practice complexities. Subsequent investigations must incorporate cross-cultural comparisons and cross-disciplinary exploration to systematically examine DGBL’s pedagogical adaptability within the TAM framework, ultimately validating and optimizing educational application frameworks across diverse learning ecosystems. Such integrated efforts would facilitate nuanced understanding of motivational drivers and contextual challenges while advancing pedagogical value validation.

## Supporting information

S1 Appendix Questionnaire. (DOCX)

S2 Appendix Data. (XLSX)
