# Supplementary material for: Exploring EFL primary school teachers’ behavioral intention towards digital game-based learning
Source: PLoS One. 2026 Apr 1;21(4):e0346229. doi: 10.1371/journal.pone.0346229 (PMC13042888; doi:10.1371/journal.pone.0346229)
Supplement: S1 Appendix — (DOCX) [file pone.0346229.s001.docx]

**S1 Appendix. Questionnaire**

**Informed Consent Form**

​Dear Teacher,​​

Thank you for considering participation in our research study. Before proceeding with this questionnaire, please carefully review the following commitments and assurances. Your involvement is invaluable, and we are committed to protecting your confidentiality and respecting your rights.

​**1. Data Confidentiality​**

We will rigorously protect the privacy of your personal information and responses. All data provided will be used exclusively for this research and analyzed/reported in anonymized form. No personally identifiable information will be disclosed to third parties without your explicit permission.

​**2. Voluntary Participation​**

Your participation is entirely voluntary. You retain the right to withdraw at any time without adverse consequences. Should you choose to withdraw, your data will be permanently deleted from the study.

​**3. Research Purpose​**

This study investigates behavioral intentions of primary school EFL teachers regarding digital game-based learning (DGBL) in classroom instruction. Your responses will critically contribute to understanding key factors influencing DGBL integration.

​**4. Data Collection​**

The questionnaire will be accessible via Wen Juan Xing from ​February 15 to March 18, 2024, with data compilation occurring between ​March 19 and April 1, 2024.

​**5. Data Usage​**

Your data will be utilized solely for academic research purposes, including analysis, scholarly publications, and supporting this study’s conclusions. All usage adheres to principles of academic integrity and complies with relevant ethical and legal standards.

​**6. Feedback​**

For questions, concerns, or requests for clarification regarding this survey, please contact: 20153429@qlnu.edu.cn​

We sincerely appreciate your time and contribution. Your insights will significantly advance our research and support progress in educational innovation.

**After carefully reviewing the Informed Consent Form above, do you voluntarily agree to participate in this study under the stated terms?​**

A. Agree

B. Disagree

**Behavioral Intention towards Digital Game-Based Learning Questionnaire**

This questionnaire consists of two sections. In multiple-choice questions, choose only one option that best describes your situation.

**Section 1**

***General information about respondents and available resources.***

1. Gender:

A. Female

B. Male

1. Age:

A. Less than 25

B. 26–30

C. 31–35

D. 36–40

E. 41–45

F. 46–50

G.50 and above

1. The number of years you have taught（Pedagogical tenure）:

A. Less than 5

B. 6–10

C. 11–15

D. 16–20

E. 21–25

F. 26–30

G. 30 and above

1. Average number of students in your class:

A. Less than 10

B.11–20

C.21–30

D.31–40

E.41–50

F.51–60

G. More than 60

1. Choose your preferred teaching approach:

A. Mainly teacher-centered (e.g., teacher in the center of the classroom, leads discussions, lectures).

B. More teacher-centered than student-centered.

C. Balance between teacher-centered and student-centered approaches.

D. More student-centered than teacher-centered.

E. Mainly student-centered (e.g., cooperative learning, discovery learning).

1. Availability of IT personnel in your educational institution (for maintenance and operation of equipment):

A. Absent

B. Available upon request

C. Always available

1. Specify how often you use Digital Game to teach students in your lessons:

A. Never

B. Rarely

C. When necessary

D. Often

E. Always

**Section 2**

**Analytical factors on ATT, PU, TP, TA, BI of the use of digital game (DB) technology in classroom.**

Using the scale, indicate the extent to which you disagree or agree with the following statements with using DB in classroom:

***Extent of Agreement:***

1. ***Strongly Disagree 2. Disagree 3. Neutral 4. Agree 5. Strongly Agree***

| **Items** |  |  |  | **Extent of Agreement** |
| --- | --- | --- | --- | --- |
|  |  |  |  | \| **1** \| **2** \| **3** \| **4** \| **5** \| \| --- \| --- \| --- \| --- \| --- \| |
| **Attitude towards Technology (ATT)** | | | | |
| AT1-Using DG technology is pleasant. | | | | \| **□** \| **□** \| **□** \| **□** \| **□** \| \| --- \| --- \| --- \| --- \| --- \| |
| AT2-I find using DG technology to be enjoyable. | | | | \| **□** \| **□** \| **□** \| **□** \| **□** \| \| --- \| --- \| --- \| --- \| --- \| |
| AT3-I have fun using DG technology. | | | | \| **□** \| **□** \| **□** \| **□** \| **□** \| \| --- \| --- \| --- \| --- \| --- \| |
| **Perceived Usefulness (PU)** | | | | |
| PU1-Using DG technology enables me to accomplish tasks more quickly. | | | | \| **□** \| **□** \| **□** \| **□** \| **□** \| \| --- \| --- \| --- \| --- \| --- \| |
| PU2-Using DG technology enhances my effectiveness. | | | | \| **□** \| **□** \| **□** \| **□** \| **□** \| \| --- \| --- \| --- \| --- \| --- \| |
| PU3-Using DG technology increases my productivity. | | | | \| **□** \| **□** \| **□** \| **□** \| **□** \| \| --- \| --- \| --- \| --- \| --- \| |
| **Technology Perception (TP)**  # Using DG in classroom … | | | | |
| TP1-Improves student academic record. | | | | \| **□** \| **□** \| **□** \| **□** \| **□** \| \| --- \| --- \| --- \| --- \| --- \| |
| TP2-Makes classroom management more difficult. | | | | \| **□** \| **□** \| **□** \| **□** \| **□** \| \| --- \| --- \| --- \| --- \| --- \| |
| TP3-Promotes the development of communication skills (e.g. writing skills, presentation skills). | | | | \| **□** \| **□** \| **□** \| **□** \| **□** \| \| --- \| --- \| --- \| --- \| --- \| |
| TP4-Requires extra resources, time and effort. | | | | \| **□** \| **□** \| **□** \| **□** \| **□** \| \| --- \| --- \| --- \| --- \| --- \| |
| TP5-Is successful only if there’s adequate teacher training in the use of DG technology in classroom. | | | | \| **□** \| **□** \| **□** \| **□** \| **□** \| \| --- \| --- \| --- \| --- \| --- \| |
| TP6-Is successful only if equipment is regularly maintained by IT personnel. | | | | \| **□** \| **□** \| **□** \| **□** \| **□** \| \| --- \| --- \| --- \| --- \| --- \| |
| TP7-Is an effective tool for students of all abilities. | | | | \| **□** \| **□** \| **□** \| **□** \| **□** \| \| --- \| --- \| --- \| --- \| --- \| |
| TP8-Effective if teachers participate in selection and implementation of DG technology. | | | | \| **□** \| **□** \| **□** \| **□** \| **□** \| \| --- \| --- \| --- \| --- \| --- \| |
| TP9-Allows to accommodate individual attributes of students. | | | | \| **□** \| **□** \| **□** \| **□** \| **□** \| \| --- \| --- \| --- \| --- \| --- \| |
| TP10-Motivates students to get more involved in learning activities. | | | | \| **□** \| **□** \| **□** \| **□** \| **□** \| \| --- \| --- \| --- \| --- \| --- \| |
| TP11-Requires DG technological training, which takes too much time. | | | | \| **□** \| **□** \| **□** \| **□** \| **□** \| \| --- \| --- \| --- \| --- \| --- \| |
| TP12-Promotes the development of student interpersonal skills. | | | | \| **□** \| **□** \| **□** \| **□** \| **□** \| \| --- \| --- \| --- \| --- \| --- \| |
| **Technology Anxiety (TA)** | | | | |
| TA1-When I consider the capabilities of technology, I think about how difficult my future will be. | | | | \| **□** \| **□** \| **□** \| **□** \| **□** \| \| --- \| --- \| --- \| --- \| --- \| |
| TA2-I have an uneasy, upset feeling when I think about technology. | | | | \| **□** \| **□** \| **□** \| **□** \| **□** \| \| --- \| --- \| --- \| --- \| --- \| |
| TA3-I feel my heart sinking when I hear about technology advancement. | | | | \| **□** \| **□** \| **□** \| **□** \| **□** \| \| --- \| --- \| --- \| --- \| --- \| |
| **Behavioral Intention (BI)** | | | | |
| BI1–I will continue to learn about DG technological knowledge. | | | | \| **□** \| **□** \| **□** \| **□** \| **□** \| \| --- \| --- \| --- \| --- \| --- \| |
| BI2–I will keep myself updated with the latest DG technological applications. | | | | \| **□** \| **□** \| **□** \| **□** \| **□** \| \| --- \| --- \| --- \| --- \| --- \| |
| BI3–I plan to spend time in learning DG technology in the future. | | | | \| **□** \| **□** \| **□** \| **□** \| **□** \| \| --- \| --- \| --- \| --- \| --- \| |
| BI4–I will pay more attention to emerging DG technological applications. | | | | \| **□** \| **□** \| **□** \| **□** \| **□** \| \| --- \| --- \| --- \| --- \| --- \| |
| BI5–I intend to use DG technology to assist my teaching. | | | | \| **□** \| **□** \| **□** \| **□** \| **□** \| \| --- \| --- \| --- \| --- \| --- \| |
